# 3D multimodal cardiac data reconstruction using angiography and computerized tomographic angiography registration

**DOI:** 10.1186/s13019-015-0249-2

**Published:** 2015-04-22

**Authors:** Rohollah Moosavi Tayebi, Rahmita Wirza, Puteri S B Sulaiman, Mohd Zamrin Dimon, Fatimah Khalid, Aqeel Al-Surmi, Samaneh Mazaheri

**Affiliations:** 1 Faculty of Computer Science and Information Technology, University Putra Malaysia, Selangor, Malaysia; 2 Cardiothoracic Unit, Surgical Cluster, Faculty of Medicine, UiTM, Selangor, Malaysia; 3 Department of Computer Engineering, Islamic Azad University, Shahr-e-Qods branch, Tehran, Iran

**Keywords:** Angiography, Computerized tomography angiography, Segmentation, Labeling, Multimodal registration, 3D reconstruction

## Abstract

**Background:**

Computerized tomographic angiography (3D data representing the coronary arteries) and X-ray angiography (2D X-ray image sequences providing information about coronary arteries and their stenosis) are standard and popular assessment tools utilized for medical diagnosis of coronary artery diseases. At present, the results of both modalities are individually analyzed by specialists and it is difficult for them to mentally connect the details of these two techniques. The aim of this work is to assist medical diagnosis by providing specialists with the relationship between computerized tomographic angiography and X-ray angiography.

**Methods:**

In this study, coronary arteries from two modalities are registered in order to create a 3D reconstruction of the stenosis position. The proposed method starts with coronary artery segmentation and labeling for both modalities. Then, stenosis and relevant labeled artery in X-ray angiography image are marked by a specialist. Proper control points for the marked artery in both modalities are automatically detected and normalized. Then, a geometrical transformation function is computed using these control points. Finally, this function is utilized to register the marked artery from the X-ray angiography image on the computerized tomographic angiography and get the 3D position of the stenosis lesion.

**Results:**

The result is a 3D informative model consisting of stenosis and coronary arteries’ information from the X-ray angiography and computerized tomographic angiography modalities. The results of the proposed method for coronary artery segmentation, labeling and 3D reconstruction are evaluated and validated on the dataset containing both modalities.

**Conclusions:**

The advantage of this method is to aid specialists to determine a visual relationship between the correspondent coronary arteries from two modalities and also set up a connection between stenosis points from an X-ray angiography along with their 3D positions on the coronary arteries from computerized tomographic angiography. Moreover, another benefit of this work is that the medical acquisition standards remain unchanged, which means that no calibration in the acquisition devices is required. It can be applied on most computerized tomographic angiography and angiography devices.

## Background

### Preliminary consideration

Among the cardiovascular system diseases, Coronary Artery Disease (CAD) is an important issue, which usually stands behind the loss of life around the world today. In fact, CAD is associated with blockage as well as narrowing the left or perhaps right coronary artery vessels. Therefore, a precise method to visualize coronary arteries is highly needed. There are several medical imaging techniques, which can be used for diagnosing heart diseases; X-ray Angiography, Cardiac Computerized Tomography Angiography (CTA), Magnetic Resonance Angiography (MRA), Cardiac Positron Emission Tomography (PET), Single-Photon Emission Computed Tomography (SPECT), Echocardiography [1] and so forth. Among these modalities, Cardiac Computerized Tomography Angiography (CT Angiography or briefly CTA), and also X-ray Angiography (X-ray arteriography or briefly angiography) are the best ways for visualizing coronary arteries.

**Figure 1:**
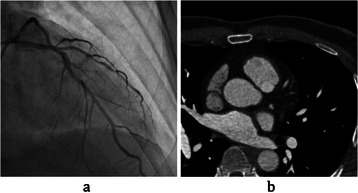
Data for “Patient3”. **(a)** An example of selected angiogram. **(b)** An example of a CTA slice.

Generally, the CT scan is a form of X-ray, which utilizes a computer system to generate cross-sectional images of the human body. The CTA is a type of medical exam that mixes a CT scan with the injection of a specific dye, referred to as a contrast material, to generate images of vessels in specific parts of the body. For this aim, the contrast material is often injected into a vein started in the hand or arm. When CTA is done, a series of images will be created, which can be observed as an axial view of cardiac components, such as cardiac chambers, aorta, heart’s muscle and coronary arteries as well. These images are referred to as CTA slices in this paper. One can get the 3D reconstruction of coronary arteries and also find out whether a plaque build-up has narrowed patient’s vessels or not. Second, modality is Angiography, which can be well used to visualize the coronary arteries and diagnose the blockage and stenosis in real-time. Therefore, most physicians prefer to use this modality instead of others to diagnose and treat cardiac coronary artery diseases. Coronary artery angiography is performed by injecting the radio-opaque contrast agent into the coronary arteries and imaging using X-ray based techniques such as fluoroscopy. A series of blood vessels radiographs is called angiograms (or angiographs). A sample of an angiogram and a CTA slice are shown in Figure 1.

### Medical context

#### Cardiovascular system anatomy

**Figure 2:**
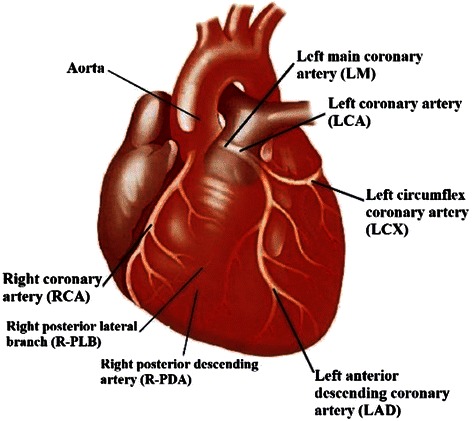
Cardiovascular system anatomy – coronary arteries.

Coronary arteries are a vital part of the cardiovascular system, which mostly relies on the surface of the heart and transports the blood to the heart muscle. As shown in Figure 2, coronary artery vessels are categorized into two principal parts: Right Coronary Artery (RCA) and Left Coronary Artery (LCA). RCA stems from the right aortic sinus and LCA stems from the left aortic sinus. The first part of the left coronary artery is referred to as the left main coronary artery (LM). This blood vessel can be about *5 mm* wide and less than *30 mm* long. LM branches directly into a pair of arteries: Left circumflex coronary artery (LCX) and left anterior descending coronary artery (LAD). LCX circles around the left side of the heart, which is embedded throughout the surface of the rear side of the heart. LAD is embedded throughout the surface of the front side of the heart. Each LCX and LAD artery bifurcates into smaller sub arteries; three septal arteries (S1 ~ S3) and three diagonal arteries (D1 ~ D3), which originate from LAD, and also two marginal arteries (OM1 and OM2), which come from LCX. The end of RCA bifurcates into a pair of smaller arteries: right posterior lateral branch (R-PLB) and right posterior descending artery (R-PDA).

#### Medical problem

Different modalities hold different information in assisting the physicians in making decision. Previously, they viewed these images in-separate or adjacent windows, and mentally fused these images together. Even though medical image registration and image fusion have been successfully implemented in other organs such as the brain and lungs, medical image registration and fusion for the heart present different challenges. First, since the heart is beating, fusion requires synchronization with the rhythm of the heart for different phases. Second, the heart is a non-solid organ, thus acquired images give a vague impression.

Precise computer-assisted coronary artery analysis is consequential in later diagnosis and treatment with cardiologists and cardiac surgeons. As explained in Section 1.1, angiography and CTA are the best modalities to visualize coronary arteries and CAD diagnosis. Individually, these modalities provide valuable information, but do not represent the complete information about coronary arteries. Thus, it is important for specialists (cardiologist or cardiac surgeon) to combine informative data in both modalities. Indeed, specialists believe that information about stenosis points in coronary arteries from angiogram, such as the 3D position, and their relative localization with respect to the corresponding segmented coronary artery from CTA is an important aid in CAD diagnosis.

There are few hybrid/combined devices like SPECT/CT, PET/CT, PET/MRI and MRI/PET, which give combined informative data. To the best of our knowledge, there no hybrid device is available for combining the results of angiography and CTA devices. Also, these devices are very expensive and none gives back the 3D position of stenosis lesion of the coronary arteries. In a successful manner, these two modalities are available in most hospitals nowadays. Therefore, one way to aid the specialist is to set up the connection between stenosis points from an angiography along with their 3D positions on the coronary arteries from CTA, so that they have a 3D informative model consisting of stenosis and coronary arteries’ information from both modalities.

### Previous work

This work introduces a new method for 3D reconstruction of stenosis point on coronary arteries through the registration of both CTA and angiography modalities. Three main phases were defined for this method: (1) Coronary artery labeling and segmentation in angiography. (2) Coronary artery segmentation and labeling in CTA, and (3) Registration of CTA and angiography images. It is worth noting that for the registration phase in this work, it is highly required for the coronary arteries to be segmented, labeled and also, their control points are extracted precisely from both modalities. The previous works of each above phases are explained as follows.

Coronary artery segmentation and labeling are crucial steps, because further processes such as 3D reconstruction, fusion with other modalities, stenosis measurement, and blood flow analysis use the result of these steps. There are some difficulties in coronary artery segmentation from angiograms; such as poor signal to noise ratio and artefacts, which caused by organs such as the backbone and ribs. Several methods have been proposed to segment coronary arteries in angiograms [2,3], and [4]. However, they only segmented coronary arteries without labelling them. Xu et al. in [5] proposed an algorithm for coronary artery centreline tracking in angiograms using matched filter on the eigenvalues. In another study, Hernández-Vela et al. [6] introduced an accurate coronary centreline extraction in angiograms. Several semi-automatic algorithms were proposed relating to the coronary artery segmentation. One of the limitations of the proposed algorithms is that they involve users in defining seed points to locate the coronary arteries. For instance, Wang et al. [7] proposed a method for coronary artery segmentation in angiograms, but it requires a seed point to start. Meanwhile, other algorithms suffer from high computational complexity. For instance, Zhou et al. [8] proposed an automatic approach for segmenting coronary arteries in angiogram. One of the limitations of their work is the time complexity, and also non-precise arteries extraction. Some other algorithms have proposed segmentation for general blood vessels. However, adopting these algorithms on the coronary artery in angiograms may lead to the appearance of some artefacts in the result. For instance, Li et al. [9] proposed a region-based active contour model for vessel segmentation. Running their algorithms on angiograms would lead to inefficient artery segmentation, whereby parts of the background might appear, while parts of the arteries disappear. Also, none of the above method labels coronary arteries based on the angiogram segmentation result. The limitations of previous algorithm motivated us to propose a new method for coronary artery segmentation and labelling, which visualizes and labels all main coronary arteries from angiography images.

Coronary artery segmentation using CTA and their 3D reconstruction should be done precisely because registration using images from angiography modality is done based on this result. However, there are plenty of problems in coronary artery segmentation and labelling from CTA slices. Firstly, coronary arteries are shown as small parts, semi-circular or tubular shape in each slice. Therefore, tracking via slices is not a straightforward process. Secondly, artefacts from other bodily organs in CTA slices, such as the backbone, ribs, cardiac chambers and other components should be removed from the final segmented image. Several algorithms have been proposed regarding coronary artery segmentation and 3D reconstruction from CTA slices [10]. Most of them used the 3D Frangi’s algorithm for coronary artery segmentation from CTA [11,12]. However, these algorithms suffer from artefacts, such as false step edge responses in some parts of the coronary arteries, especially near the right atrium. Yang et al. [13] solved this problem by proposing an improved 3D Frangi’s method. They improved the 3D Frangi’s vesselness filter by adding local geometrical features. Another drawback of the current methods is their non-capability of labelling, especially at the same time with the segmentation phase. The only study on coronary artery labelling from CTA was proposed in [14], where arteries are labeled after segmentation in a different phase. They identified the main branches using point-set registration method as proposed in [15]. However, the registration phase of their algorithm suffers from high computational complexity and also, the control points required for registration with angiogram in our work are not extracted. These mentioned limitations motivated us to propose a new method for coronary artery segmentation, labelling and also 3D reconstruction of CTA slices.

As explained before, the aim of this work is 3D reconstruction of stenosis point through CTA and angiography image registration. Therefore, registering both CTA and angiography modalities is one of the important phases of this work. Several works have been done for coronary artery registration in these two modalities. Some of them were done for 2D/3D coronary artery registration in both modalities [16-18], and some proposed 2D/2D registration to guide endovascular stent grafting [19,20], and [21]. Also, another work on coronary artery registration was done in [22], but specific angiography devices (biplane) were needed in their work. Furthermore, all of the above studies proposed registration algorithms to align coronary arteries in both modalities CTA and angiography. The benefit of these works is the usage of CTA result (pre-interventional/pre-operative) as the image guidance in angiography (interventional/intra-operative) procedure for percutaneous coronary intervention (PCI). However, these methods cannot be applied in our work because they only aligned some correspondent arteries together, and therefore, it is not possible to use them for registering the start, bifurcations and end points of the correspondent coronary arteries from two modalities. The benefit of having these points is it enables the estimation of stenosis point between the nearest two points from one artery in angiogram to the correspondent artery in CTA. The above mentioned limitations motivated us to propose a new method for feature-based coronary artery registration using proper control points.

## Methods

**Figure 3:**
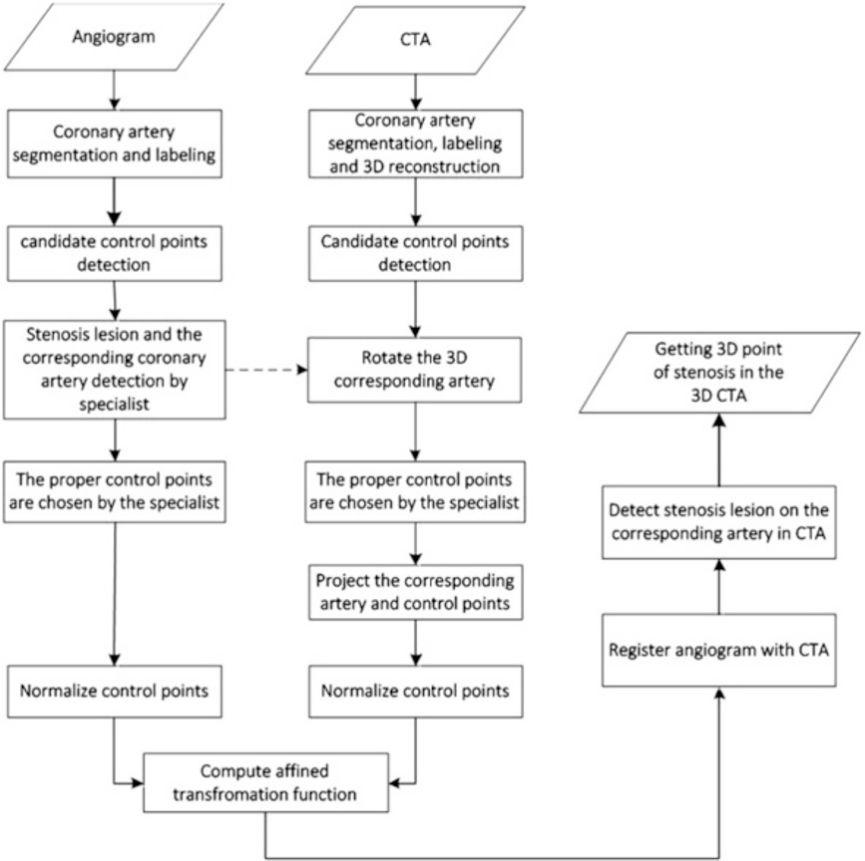
Flowchart for the proposed method for coronary artery registration and 3D stenosis point detection.

The key-ideas of the proposed method are: (1) No calibration process required for the input images. (2) No need for 3D reconstruction images from 2D angiography. (3) No need for multiple views of angiography. (4) No need for specific angiography (like biplane) devices. Figure 3 provides a diagram outlining all steps of the proposed method.

Some steps in the proposed method can be described as follows.

- The specialist chooses an angiogram such that the stenosis lesion can be observed clearly.
- 2D main coronary artery segmentation and labeling for the chosen angiogram are done in 2D (*u*^'^, v^'^) plane.
- 3D main coronary artery segmentation and labeling are done for CTA modality in (*x,y,z*) Cartesian coordinate system.
- Candidate control points are detected automatically for both modalities.
- Stenosis lesion is marked in angiogram by the specialist. In addition, the coronary artery with the stenosis is chosen for registration.
- CTA is rotated to each direction (*x,y,z*) to get an almost similar view of the artery as displayed in the angiogram.
- Correspondent control points are marked by the specialist in both angiogram and CTA modalities.
- Rotated artery and their control points in CTA are projected onto 2D (*u*, v) plane.
- The selected control points are normalized for both modalities, individually.
- Affine transformation function is computed for the normalized control points.
- The correspondent coronary arteries from both modalities in (*u*^'^, v^'^) and (*u*, v) planes are registered.
- Stenosis lesion from the angiogram is detected on the correspondent artery from CTA.
- Stenosis point is back projected in the 3D Cartesian coordinate system (*x,y,z*).

The crucial steps of the proposed method are: (1) Coronary artery segmentation and labelling in angiogram. (2) Coronary artery segmentation and labelling in CTA. (3) Feature-based registration of coronary arteries in CTA and angiogram. These steps are respectively detailed as follows.

### Coronary artery segmentation and labelling in angiogram

In this section, a new method for coronary artery labelling and segmentation from angiogram is proposed. The methodology of this work includes five phases, as described in the following subsections. In the first phase, after removing noise from the raw angiograms with Discrete Wavelet Transform (DWT), all arteries are sharpened with Starlet Wavelet Transform (SWT). In the next step, the main coronary arteries are segmented by applying the modified SWT. After that, coronary artery centrelines are segmented, and also detached from each other. Then, all centrelines are labeled, and finally, coronary arteries are labelled by constructing a proper mask.

#### Angiogram pre-processing

Each type of X-ray angiography has a moving DICOM format result. Dealing with this type of imaging is too difficult. First, the angiograms have to be converted into *n* 2D bitmap frames {*f*_1_, *f*_2_, … *f*_*n*_} on each DICOM movie angle, such that n varies from 40 to 70 frames. One optimum frame (*f*_*optimum*_) must be chosen from these frames. The optimum frame is defined as a well X-ray injected frame that the whole coronary arterial tree is contrasted with that, and also the stenosis can be detected by specialist. After choosing an appropriate frame, it is converted from RGB format to grayscale, which we call as the original input image ${I}_A^o$ for next steps.

Since angiograms suffer from a large amount of noise, the first step is noise removal. The aim of the noise removal process is to eliminate all noise while preserving the quality of the images. Here, quality hints to retain the arteries in the angiograms. When we used the traditional algorithms for removing noise in angiograms, such as the smoothing method, some arteries disappeared. Hence, another technique for removing noise should be defined by converting images into a transformation domain, such as wavelet, and then compared the transformation’s coefficients to a proper threshold value. In this way, the arteries’ structure is kept. Therefore, DWT with wavelet type Haar and level 3 was used for removing noise from angiograms in this work. The details of using DWT for noise removal are well defined in [3,23]. This step is shown in Eq (1).

1 $$ {I}_A^o\overset{denoising}{\to }{I}_A^d $$

**Figure 4:**
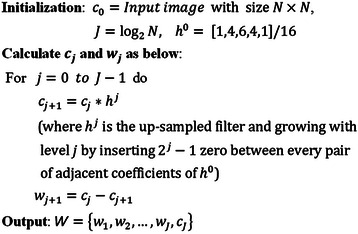
Starlet wavelet transform (SWT) algorithm [28].

The next step in pre-processing is coronary artery sharpening. In this step, we intended to sharpen the edges of arteries and erased the surrounding background using SWT, which has been defined in [3]. Because this work concentrated on the main coronary artery labelling, this method was modified. SWT, or Isotropic Undecimated Wavelet Transform (IUWT), is well known in the field of biology [24], astronomy [25] and nowadays in medical applications [3] and [26]. SWT decomposes an image *I* into *w*_*j*_, as a wavelet coefficient and *c*_*j*_ as a scale coefficient in each iteration *j* [27]. A preliminary algorithm for SWT [28] is presented in Figure 4.

**Figure 5:**
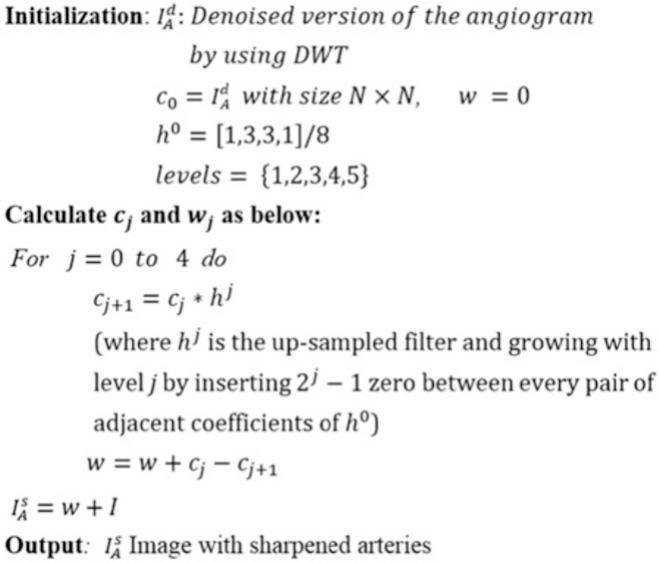
The modified starlet wavelet transform (SWT) algorithm for sharpening main coronary arteries in angiograms.

In this section, the SWT algorithm is modified to obtain sharper coronary arteries in angiograms, and in the next step, it is changed again to segment the main coronary arteries from angiograms. For segmenting the main coronary arteries from angiograms, different filters were applied with various wavelet levels (*l*) and finally, we found that using *h*^0^ = [1,3,3,1]/8 on the input image with selective wavelet levels *l* = {1,2,3,4,5} were best for sharpening. The modified algorithm for sharpening main coronary arteries in angiogram is illustrated in Figure 5.

#### Main coronary artery segmentation

**Figure 6:**
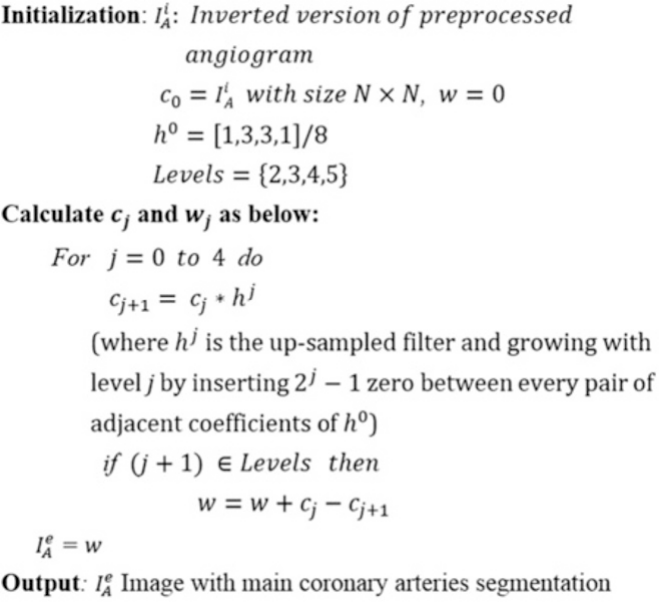
Our proposed algorithm for main coronary artery segmentation in the angiogram.

SWT is a type of wavelet transform, which is fast in computation and can be used for object segmentation [3]. In this phase, a new method for main coronary artery segmentation is proposed based on SWT application. Since intensity values of objects must be higher than background when segmenting with SWT, ${I}_A^s$ should be inverted first. The output is called ${I}_A^i$. Then, the modified SWT is applied on ${I}_A^i$ for coronary artery segmentation. Generally, applying various filters and also several wavelet levels (*l*) in SWT returns different results. In the case of angiogram, thinner arteries are shown in smaller *l* values and thicker ones in higher *l* values. In addition, using various filters returns different qualities of coronary arteries. Empirically, it was found that using wavelet levels *l* = {2,3,4,5} applying filter *h*^0^ = [1,3,3,1]/8 on ${I}_A^i$ was best for main coronary artery segmentation. The proposed algorithm is shown in Figure 6.

**Figure 7:**
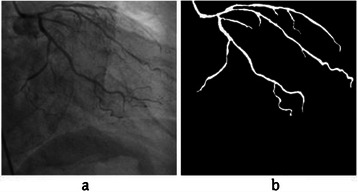
The result of main coronary artery segmentation. **(a)** A sample of input angiogram. **(b)** The result of main coronary artery segmentation from angiogram using the proposed method.

After the main coronary artery segmentation, a post-processing step was considered, which includes thresholding, followed by length refinement and filling holes, as discussed in [3]. The final result is shown in Figure 7.

#### Centreline extraction and detachment

**Figure 8:**
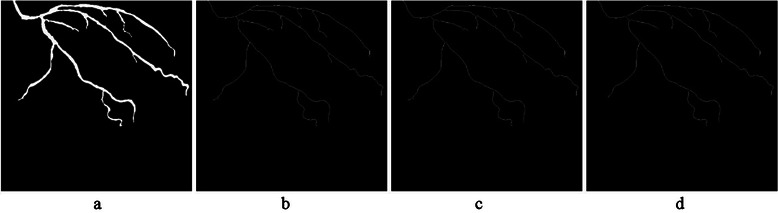
The result of coronary artery centreline extraction and detachment. **(a)** A sample of the main coronary artery segmented from an angiogram. **(b)** Centreline extraction. **(c)** Removal of branch points to achieve centreline detachment. **(d)** Removal of short segmented arteries.

In this phase, the centrelines of coronary arteries are extracted using morphological operation on the results of the previous phase ${I}_A^e$. This process removes pixels, so that all arteries are thinned to a minimally connected stroke. Then, arteries are detached. To do this, the start, the branch and the end points of arteries are detected by counting pixels’ neighbours. This process is done by convolving the centreline of the coronary arteries with a 3 by 3 kernel of ones and the results are saved in matrix ${I}_A^b$. Then, centreline detachment is achieved after removing the branch points. In addition, short segmented arteries are removed by counting pixels in each detached centreline arteries and by using a proper threshold. The result of centreline extraction is saved in ${I}_A^c$ and all starting, branching and ending points of arteries are saved separately in matrix ${I}_A^p$. These steps are shown in Figure 8 for one selected angiogram.

#### Coronary artery centreline labeling

**Figure 9:**
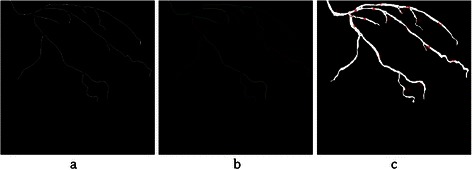
The result of centrelines labeling $\left({I}_A^{cl}\right)$. **(a)** Apply Euclidean distance transform to clear branch points. **(b)** Display each connected centreline with a different color. **(c)** Show labels as texts in the original angiogram.

Before centreline labeling, all branch points are cleared. This process is done by computing the Euclidean distance to remove some centreline pixels in ${I}_A^c$, which are closer to the branch points than to the non-vessel points. Then, centrelines are labelled by saving any connected centreline pixels with a different number in a new matrix ${I}_A^{cl}$, which has the same size as the original angiogram. The result of centreline labelling is illustrated in Figure 9.

#### Coronary artery labeling

As explained before, all centrelines of the coronary arteries are detached and saved with unique numbers in matrix ${I}_A^{cl}$. The aim of this phase is to label the coronary arteries from the labeled centrelines. First, the artery is selected by choosing the correspondent numbers in the labeled centrelines image ${I}_A^{cl}$. The output is called ${I}_A^{cs}$. Then, a proper mask is constructed to segment the correspondent artery in ${I}_A^e$. Mask construction is done by computing the Euclidean distance transforms between all pixels on the selected labeled centrelines ${I}_A^{cs}$ and the boundary of the correspondent artery in ${I}_A^e$. Then, the maximum value of these distances is chosen as the mask radius *r*. After that, all pixels on the selected centrelines in ${I}_A^{cs}$ are inflated with radius *r*. to achieve the proper mask. This mask is applied on ${I}_A^e$ to construct the labelled coronary artery and the result is saved in *I*_*A*_. The start, the end and the branch points on the selected artery are obtained using Eq (2).

2 $$ {P}_A={I}_A\cap {I}_A^p $$

**Figure 10:**
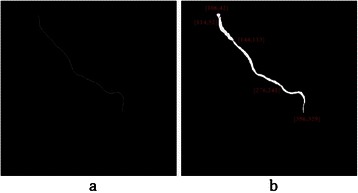
The result of coronary artery labeling for centreline numbers 3, 5, 9 and 15 of the input angiogram. **(a)** Selected labelled centrelines $\left({I}_A^{cs}\right)$. **(b)** Labeled coronary artery constructed (***I***_***A***_) with candidate control points (***P***_***A***_).

*P*_*A*_ is the candidate control points in the angiogram and is used for the registration part of this work. The result of the main coronary artery labelling for one sample artery is shown in Figure 10. The candidate control points *P*_*A*_ are also displayed on the start, the branch and the end points of the labeled artery.

### Coronary artery segmentation, labelling and 3D reconstruction from CTA

**Figure 11:**
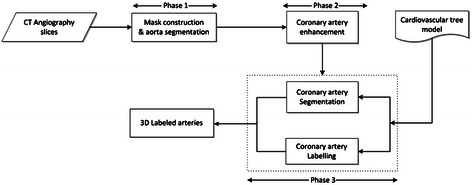
The general schema of the proposed method for coronary artery labeling in CTA.

In this part, a new method for coronary artery segmentation, labelling and 3D reconstruction from CTA slices proposed. Figure 11 displays this method, which consists of three main phases: 1) mask construction and aorta segmentation, 2) coronary artery enhancement, 3) coronary artery segmentation and labelling.

The details of every phase are described in the next subsections.

#### Mask construction and aorta segmentation

**Figure 12:**
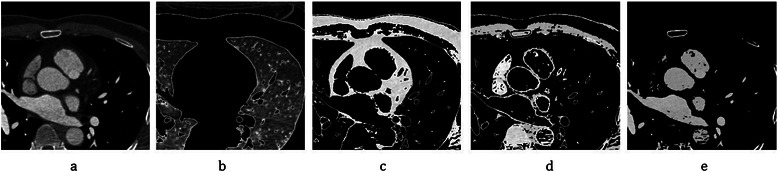
CTA data categorization based on Hounsfield Unit (*HU*). **(a)** A selected CTA slice as an input. **(b)** Region 1 containing pulmonary tissues. **(c)** Region 2 including the diaphragm and pericardium. **(d)** Region 3 including uncontrasted coronary arteries, myocardium, and hepatica tissues. **(e)** Region 4 including contrasted coronary arteries, ventricles, bone, aorta, pulmonary vessels.

In the first phase, CTA slices are classified anatomically. Normally, every CTA slice is decomposed into these main components: pulmonary tissues, pulmonary vessels, aorta, diaphragm, pericardium, myocardium, aorta, bones, ventricles, hepatica tissues and (contrasted/uncontrasted) coronary arteries [29]. To distinguish these components, Hounsfield Unit (*HU*) is used, which is computed by voxel intensity. It is given by:

3 $$ HU=\left( Pixel\ value* Slope\right)+ Intercept $$

where *Slope* and *Intercept* parameters are obtained from the DICOM info of every CTA device. In this work, the histograms of 12 CTAs were assessed and it was found that most of them had four main regions through “-1024” to “+1000” (*HU*) values, which specify different types of heart’s components. As result of this experiment, it is shown *HU* range for every component is different. For some such as contrasted coronary arteries, bone, aorta and ventricles, it is intrinsically high (between “+391” and “+1000” in region 4), while for others such as pulmonary tissue, it is markedly low because of the air (between “-1024” to “-225” in region 1). For particular soft tissues such as the diaphragm and pericardium tissues, the *HU* range is regarded as “-226” to “+30” as considered in region 2, and for uncontrasted coronary arteries, myocardium and hepatica tissues, it is between “+31” and “+390”, which fits in region 3. The result of the categorization is illustrated in Figure 12.

**Figure 13:**
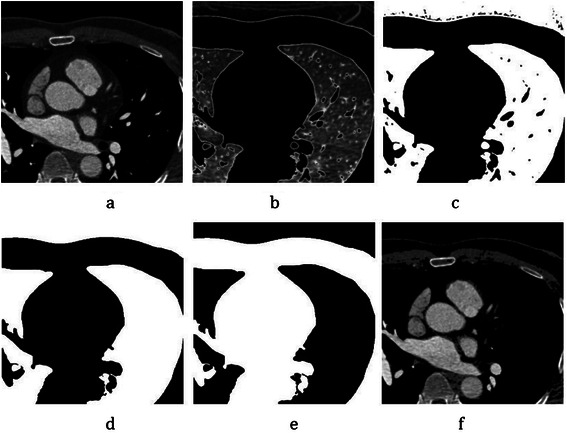
Mask construction for CTA images. **(a)** Select CTA slice. **(b)** Extract region 1 based on Hounsfield Unit (*HU*) measurement. **(c)** Create the binary image by considering specific threshold. **(d)** Fill holes (for pulmonary vessels). **(e)** Construct mask. **(f)** Apply mask on regions 3 and 4 (*HU* ≥ 31).

Since the most important component in this work is the coronary artery, first, we categorized each CTA slice as it has the most probably coronary arteries through various other heart components. Therefore, we focused on regions 3 and 4 because the coronary arteries (both contrasted and uncontrasted) are shown in these two regions. Due to the fact that pulmonary vessels and tissues are located near to the coronary arteries in some slices, especially bordering the heart, and could be considered as coronary arteries incorrectly in some slices, we constructed a mask from region 1 to remove them in all slices. First, region 1 is extracted using *HU*. values for every CTA slice and then, the specific threshold is considered to create binary images. After that, the filling hole method is applied to remove the holes coming from the pulmonary vessels. Finally, the mask is constructed by reversing the black and white colors. These steps are shown in Figure 13 for a selected CTA slice.

**Figure 14:**
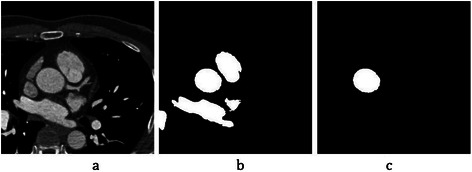
Aorta segmentation in initial slices. **(a)** A selected slice from the initial CTA slices. **(b)** Extraction of region 4 based on Hounsfield Unit (*HU*) measurement and also pre-processing steps for removing small objects. **(c)** The result of aorta segmentation by applying Hough circle transform and performing some post-processing steps.

Prior to coronary artery enhancement, aorta segmentation is done using Hough circle transform in initial axial slices. First, region 4 is selected based on *HU* measurement, small objects such as noise are removed. Then, the Hough circle transform is applied to detect circular objects and also some post-processing methods are employed to remove residual noise and fill small holes. It is shown for a selected slice in Figure 14. Finally, the aorta is saved in a 3D matrix named *M*_*Aorta*_ for all initial slices by considering the correspondent slice numbers as the rows for the 3D matrix.

#### Coronary artery enhancement

In the second phase, Frangi multi-scale filter is applied to measure the vesselness components based on the eigenvalues in the Hessian matrix [30]. This method is used to find and enhance tubular components by simply computing the second order derivatives in the Gaussian kernel at various scales and giving a value between *0* and *1* for each pixel x at certain scale *σ*. This vesselness measure function is formulated in Eq (4).

4 $$ v\left(\mathrm{x},\sigma \right) = \left\{\begin{array}{c}\hfill 0\kern14em ,\ if\ {\lambda}_1<0\hfill \\ {}\hfill exp\left(-\frac{{\mathrm{\mathcal{R}}}_B^2}{2{\varphi}_1^2}\right)\left(1- exp\left(-\frac{s^2}{2{\varphi}_2^2}\right)\right)\kern1.25em ,\ otherwise\hfill \end{array}\right. $$

where ${\mathrm{\mathcal{R}}}_B=\frac{\left|{\lambda}_1\right|}{\left|{\lambda}_2\right|}$., $s = \sqrt{{\lambda_1}^2+{\lambda_2}^2}$. and *λ*_1_, *λ*_2_ are eigenvalues in a 2D Hessian matrix. *φ*_1_. and *φ*_2_ determine the level of sensitivity in the filter to the amounts ℛ_*B*_ and *S*, respectively.

The 2D Hessian matrix for a given pixel x and scale *σ* is also given by:

5 $$ {H}_{\sigma}\left(\mathrm{x}\right)=\left[\begin{array}{cc}\hfill {I}_{xx}\left(\mathrm{x}\right)\hfill & \hfill {I}_{xy}\left(\mathrm{x}\right)\hfill \\ {}\hfill {I}_{yx}\left(\mathrm{x}\right)\hfill & \hfill {I}_{yy}\left(\mathrm{x}\right)\hfill \end{array}\right] $$

where *I*_*αβ*_(x) indicates the second order derivative of the input image at pixel x obtained by convolving the image using the 2D Gaussian kernel G(,*s*) at scale *σ*. These definitions are formulate in Eq (6) and (7).

6 $$ {I}_{\alpha \beta}\left(\mathrm{x}\right)=I*{\sigma}^2\frac{\partial^2G\left(\mathrm{x},\sigma \right)}{\partial_{\alpha }{\partial}_{\beta }} $$

7 $$ G\left(\mathrm{x},\sigma \right)=\frac{1}{2\pi {\sigma}^2}{e}^{-\frac{\parallel \mathrm{x}{\parallel}^2}{2{\sigma}^2}} $$

**Figure 15:**
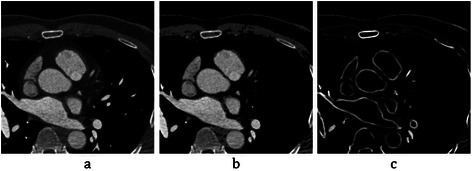
Coronary artery enhancement. **(a)** Selected slice of the CTA. **(b)** Apply the mask. **(c)** The result of enhancement algorithm.

After applying the mentioned filter, the coronary arteries are enhanced as shown in Figure 15.

#### Coronary artery segmentation and labeling from CTA

**Figure 16:**
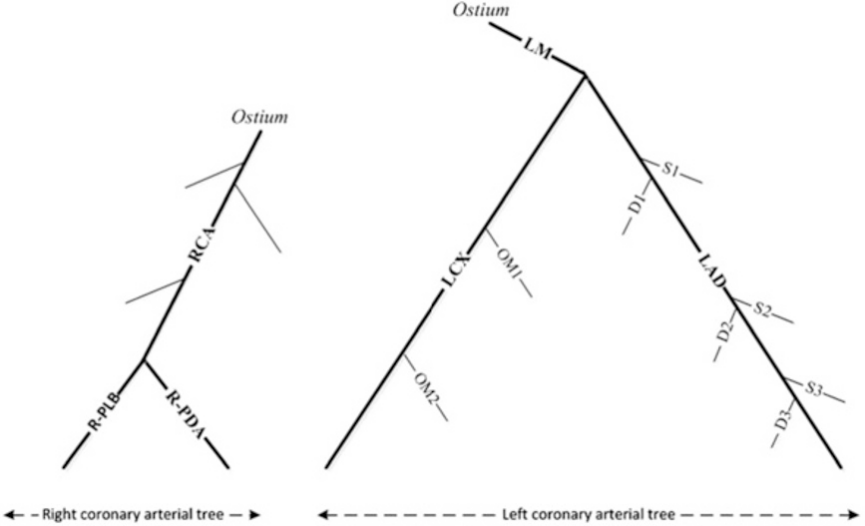
The cardiovascular tree model.

As shown in Figure 15, there are many components that should be removed to obtain only the coronary arteries. For this, the Intersection Tracking method is proposed for tracking coronary arteries through 2D slices from ostium to the end. Before discussing the proposed method, the “Cardiovascular tree model” is defined based on Figure 2. As shown in Figure 16, LCA and RCA have a tree-like structure and can be categorized as the left coronary arterial tree and right coronary arterial tree. We called this structure as the “Cardiovascular tree model”, which is used in this work as a prior knowledge for coronary artery labelling.

**Figure 17:**
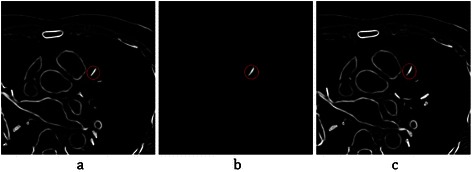
Continuous pixels of the LAD artery in three sequential slices. **(a)** Slice ***S***_i − 1_, before segmentation. **(b)** Segmentation of a part of the coronary artery in slice ***S***_i_. **(c)** Slice ***S***_i + 1_, before segmentation.

Before explaining the proposed algorithm, the Intersection Tracking method is explained, which is proposed for coronary artery segmentation and labelling from CTA. This method works based on the fact that each coronary artery has continuous pixels through slices from the start to the end point. Therefore, each part of the artery in every slice has intersection with the prior and the next parts in the previous and the next slices, respectively. See Figure 17 for LAD artery in three sequential slices.

**Figure 18:**
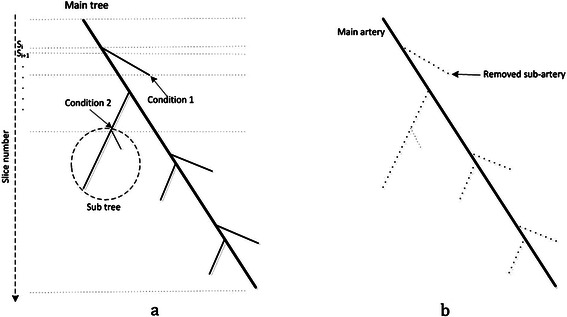
An illustration of coronary arteries’ bifurcations in the cardiovascular tree model. **(a)** Cardiovascular tree model with some bifurcations by considering two conditions. **(b)** The expected result after running the Intersection Tracking method (removed sub arteries are shown as dashed lines).
  2. One of the vessels bifurcates before another one ends: If one of the arteries bifurcates again before another one ends, it means that most probably, this artery is as the main artery and the other one should be removed. But as shown in Figure 18, sometimes, the sub-arteries, such as septal or diagonal, make a subtree by bifurcating themselves. Therefore, whenever an artery is bifurcated, new 3D and 2D matrices as mentioned above are constructed for new branches. The same procedure is done for all branches in parallel to find the main artery and remove the other ones. The bifurcation points are reserved in the proper position in 3D matrix *M*^*p*^, as explained before.
- **Step 8**: Go to step 4 for the next slice.
- **Step 9**: The centroid point *c*_*i*_. of the segmented region in matrix *H* is computed. Then, the value “1” is allocated to the *i*th row from top to bottom of 3D matrix *M*^*p*^ and 2D position *c*_*i*_. This point is used as the end point of the coronary artery.
- **Step 10**: Save the 3D matrix M as *M*_*LM*_, *M*_*RCA*_, *M*_*LAD*_ and *M*_*LCX*_, based on the coronary artery considered for segmentation in this phase. The candidate (start, bifurcation and end) points for each main coronary arteries are saved in one of the 3D matrices $$ {M}_{LM}^p, $$$$ {M}_{RCA}^p, $$$$ {M}_{LAD}^p $$ and $$ {M}_{LCX}^p, $$ as well.

As shown in the figure, the selected part of LAD has intersecting pixels with another part of LAD in the previous and next slices. Therefore, if it is possible to segment any part of the artery in specific slice *i*, other parts can be tracked and accessed through the previous and next slices. Based on this fact, and also the “Cardiovascular tree model” in Figure 16, the Intersection Tracking method is defined as follows:

- **Step 1**: Start from a seed point preserved in slice *S*_*i*_; *i* is the number of slices and can be defined based on the artery considered for segmentation. We will discuss about the seed point for each artery later in this section.
- **Step 2**: Segment the correspondent region and preserve it at the same position in a 2D temporary matrix called *H*. The size of matrix *H* is considered to be the same as slice *S*_*i*_.
- **Step 3**: Construct a 3D matrix called *M*, and allocate the *i*th row from top to bottom by the 2D matrix *H*. To preserve the start, the bifurcation and the end points of the artery, at the first centroid point *c*_*i*_ of the segmented region in matrix *H* should be computed. Then, a new 3D matrix called *M*^*p*^ is constructed, and the value “1” is allocated to the *i*th row from top to bottom and 2D position *c*_*i*_ of this matrix. This point is later used as the start point of the artery in the registration phase. Indeed, the 3D matrix *M*^*p*^ preserves the candidate control points of the coronary arteries (such as, start, bifurcation and end points)
- **Step 4**: Proceed to the next axial slice *S*_*i* + 1_ by increasing the counter *i*. Then, intersect it with the previous segmented region, which is currently saved in matrix *H*, to find the new segmented region. Plenty of segmented components are in slice *S*_*i* + 1_. Based on the Intersection Tracking method, the component that has intersection with matrix *H* is selected. If there is no intersection between matrix *H* and slice *S*_*i* + 1_, go to step 9.
- **Step 5**: Replace matrix *H* with the new segmented region in slice *S*_*i* + 1_, and allocate it in the next (*i* + 1)^th^ row in the 3D matrix M.
- **Step 6**: Bifurcation detection is done in this step. As shown in the “Cardiovascular tree model”, each main artery such as LM, LCX, LAD and RCA has some sub-arteries. Therefore, it should be examined if the artery is bifurcated in the current slice or not. If it is, the sub-artery is removed to get only the main coronary artery. The thinning process is applied on the current segmented region, which is currently saved in matrix *H*, using morphological operation and convolution with the 3 by 3 kernel of ones to check the mentioned condition. If this region includes a bifurcation, the algorithm proceeds to the next step, and value “1” is allocated for the *i*th row from top to bottom of 3D the matrix *M*^*p*^ as a bifurcation position. Else, return to step 4 for the next slice.
- **Step 7**: For discovering the continuing branch from the main artery and the sub-artery, every branched vessels are segmented and saved in two new temporary 2D matrices, *N*^'^ and *N*^' '^ (at the same position in the corresponding slice *S*_i + 1_). Also, two 3D temporary empty matrices, *M*_1_ and *M*_2_ are constructed and 2D matrices, *N*^'^ and *N*^' '^ are added on (*i* + 1)^th^ row of each of them, respectively. Then next slice is considered by increasing the counter *i*. for each branched vessel regions in parallel, which is currently saved in two temporary 2D matrices *N*^'^ and *N*^' '^, and they are tracked in the following slices using the mentioned Intersection Tracking method. This procedure continues and new segmented regions *N*^'^ and *N*^' '^ are added into the two 3D temporary matrices *M*_1_ and *M*_2_, until one of conditions appear:
  1. One of vessels reaches the end: As shown in Figure 18, the ended vessel is a sub-artery and should be removed. The correspondent 3D matrix is removed and the remaining temporary 3D matrix (*M*_1_ or *M*_2_ based on which one is considered as continuous pixels of the main artery) is merged into a main 3D matrix M. Matrix *H* is also replaced with the last region in *N*^'^ or *N*^' '^ (based on the selected part, which is considered as the main artery).

**Figure 19:**
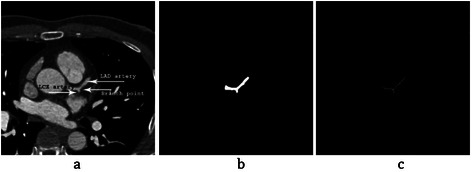
LAD and LCX seed point detection. **(a)** A CTA slice includes LM artery. **(b)** Segmented region currently saved in matrix *H*. **(c)** Thinning by means of morphological operation for bifurcation detection.

As mentioned in step 1, the Intersection Tracking algorithm starts from a seed point for each LM, LAD, LCX and RCA arteries. Different conditions are considered for choosing them. As shown in the “Cardiovascular tree model” in Figure 16, LCA is started from LM artery. Axial ices are sought from top to bottom to find slice *S*_*i*_ as LM’s seed point, where LCA starts from the aorta and *i* is the number of slice. Then, the above Intersection Tracking algorithm is applied on LM segmentation. When a bifurcation is detected in step 6, it means that the LM ends in that slice and both LAD and LCX would start from that point. Therefore, the bifurcation point is selected as a seed point for LAD and LCX segmentations, as shown in Figure 19.

**Figure 20:**
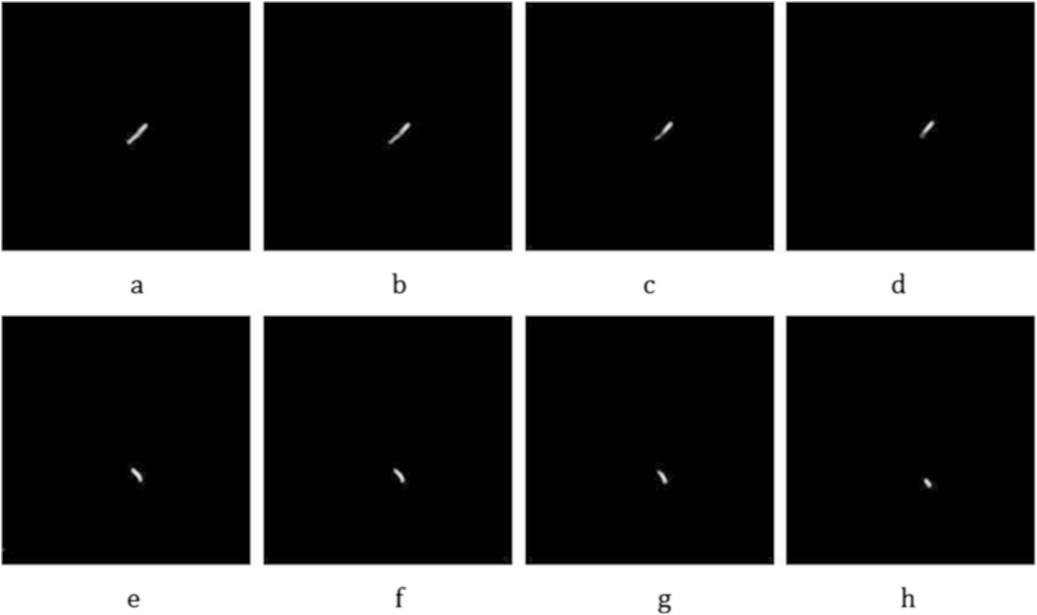
Coronary artery segmentation in each CTA slice using the proposed intersection tracking method. **(a)**-**(d)** Example of LAD segmentation. **(e)**-**(h)** Example of LCX segmentation.

For RCA, axial slices are sought from top to bottom to find slice *S*_*i*_ as RCA’s seed point, where the RCA starts from the aorta. Then, the same Intersection Tracking method is used for RCA segmentation. As shown in the “Cardiovascular tree model”, RCA has one main branch with some sub-arteries and also two main branches, called R-PDA and R-PLB. The sub arteries should be removed, except for R-PDA and R-PLB because these two sub-arteries are considered as the main parts of RCA, and sometimes, stenosis can appear in these parts. A proper threshold is defined in step 7 of the above algorithm for preserving these two sub-arteries. The steps for LAD and LCX segmentation in few sequential slices are shown in Figure 20.

The pseudo code of the proposed method in this phase is described in the following.

### Feature-based CTA and angiogram registration

As analyzed in other papers dealing with cardiac image registration [31-33], there is no previous work on coronary artery registration from CTA and angiography modalities to create a 3D reconstruction of stenosis point. In this section, the defined control points and a proper transformation function are used to register the coronary arteries from both CTA and angiography modalities.

First, different angiograms from several angles are examined by a specialist (cardiologist or cardiac surgeon) to find the best view, in which stenosis can be visualized and detected. Then, the coronary artery (which has stenosis on it) is segmented and labeled from the selected angiogram using the proposed algorithm in section 2.1, and the candidate control points are detected. Then, the position of the stenosis point is marked by the specialist. After that, the correspondent artery and their candidate control points are segmented from CTA using the proposed algorithm in section 2.2. Since the CTA results are preserved as 3D points in one of the 3D matrices *M*_*LM*_, *M*_*RCA*_, *M*_*LAD*_ or *M*_*LCX*_ (based on which one is detected as having the stenosis), it is possible to rotate the artery so that it is in the same angle as the selected angiogram. Then, the rotated CTA is projected from (*x*, *y*, *z*) Cartesian coordinate (3D) to the (*u*, *v*) plane (2D). The same projection onto the (*u*, *v*) plane is done for the candidate control points in 3D matrices $$ {M}_{LM}^p, $$$$ {M}_{RCA}^p, $$$$ {M}_{LAD}^p $$ and $$ {M}_{LCX}^p, $$ based on the selected artery. The result of the candidate control points projection from CTA is called *P*_*C*_.

Let us consider *I*_*C*_ and *I*_*A*_ as two 2D images containing the segmented correspondent arteries from the projected CTA and the angiogram, respectively. Also, *C* and *C*^'^ denote the correspondent control points selected from *P*_*C*_ and *P*_*A*_ (defined in Eq (2)), respectively.

8 $$ \begin{array}{ccc}\hfill c=\left[\begin{array}{c}\hfill \begin{array}{cc}\hfill {u}_1\hfill & \hfill {v}_1\hfill \\ {}\hfill {u}_2\hfill & \hfill {v}_2\hfill \end{array}\hfill \\ {}\hfill \begin{array}{cc}\hfill \cdot \hfill & \hfill \cdot \hfill \\ {}\hfill {u}_n\hfill & \hfill {v}_n\hfill \end{array}\hfill \end{array}\right],\hfill & \hfill {c}^{\prime }=\left[\begin{array}{c}\hfill \begin{array}{cc}\hfill {u}_1^{\prime}\hfill & \hfill {v}_1^{\prime}\hfill \\ {}\hfill {u}_2^{\prime}\hfill & \hfill {v}_2^{\prime}\hfill \end{array}\hfill \\ {}\hfill \begin{array}{cc}\hfill \cdot \hfill & \hfill \cdot \hfill \\ {}\hfill {u}_n^{\prime}\hfill & \hfill {v}_n^{\prime}\hfill \end{array}\hfill \end{array}\right]\hfill & \hfill \left(n\ge 3\right)\hfill \end{array} $$

By considering *i* as a point number, *C*_*i*_ and ${C}_i^{\prime }$ denote the correspondent control points, which is mathematically formulated in Eq (9).

9 $$ \left\{{C}_i\leftrightarrow {C}_i^{\prime}\right\} $$

In the first step of the registration, *C*_*i*_ and ${C}_i^{\prime }$ are normalized to improve the accuracy of the result. The normalization comprises of translating and scaling the coordinates of *I*_*C*_ and *I*_*A*_ images, defined as the following steps:

1. First, the control points’ coordinates inside every image are separately translated in order to bring the centroid toward the origin, formulated as follows:

10 $$ \begin{array}{ccc}\hfill {C}_T=\left[\begin{array}{c}\hfill \begin{array}{cc}\hfill {u}_1-\overline{u}\hfill & \hfill {v}_1-\overline{v}\hfill \\ {}\hfill {u}_2-\overline{u}\hfill & \hfill {v}_2-\overline{v}\hfill \end{array}\hfill \\ {}\hfill \begin{array}{cc}\hfill \cdot \hfill & \hfill \cdot \hfill \\ {}\hfill {u}_n-\overline{u}\hfill & \hfill {v}_n-\overline{v}\hfill \end{array}\hfill \end{array}\right],\hfill & \hfill {C}_T^{\prime }=\left[\begin{array}{c}\hfill \begin{array}{cc}\hfill {u}_1^{\prime }-{\overline{u}}^{\prime}\hfill & \hfill {v}_1^{\prime }-{\overline{v}}^{\prime}\hfill \\ {}\hfill {u}_2^{\prime }-{\overline{u}}^{\prime}\hfill & \hfill {v}^{\prime }-{{\overline{v}}^{\prime}}_2\hfill \end{array}\hfill \\ {}\hfill \begin{array}{cc}\hfill \cdot \hfill & \hfill \cdot \hfill \\ {}\hfill {u}_n^{\prime }-{\overline{u}}^{\prime}\hfill & \hfill {v}_n^{\prime }-{\overline{v}}^{\prime}\hfill \end{array}\hfill \end{array}\right]\hfill & \hfill \left(n\ge 3\right)\hfill \end{array} $$

In which $$ \overline{u}=\frac{{\displaystyle {\sum}_{i=1}^n}{u}_i}{n}, $$$$ \overline{v}=\frac{{\displaystyle {\sum}_{i=1}^n}{v}_i}{n}, $$$$ {\overline{u}}^{\hbox{'}}=\frac{{\displaystyle {\sum}_{i=1}^n}{u_i}^{\hbox{'}}}{n}, $$$$ {\overline{v}}^{\hbox{'}}=\frac{{\displaystyle {\sum}_{i=1}^n}{v_i}^{\hbox{'}}}{n}. $$

And the centroid points for each modality are defined as: $$ c=\left[\begin{array}{cc}\hfill \overline{u}\hfill & \hfill \overline{v}\hfill \end{array}\right] $$ and $$ {c}^{\hbox{'}}=\left[\begin{array}{cc}\hfill {\overline{u}}^{\hbox{'}}\hfill & \hfill {\overline{v}}^{\hbox{'}}\hfill \end{array}\right] $$

In the next step, *C*_*T*_ and ${C}_T^{\hbox{'}}$ are scaled so that the root mean squared (RMS) distance from the origin is equivalent to $\sqrt{2}$. It is formulated for *C*_*T*_ as:

11 $$ S\mathsf{x}RMS=\sqrt{2} $$

The RMS, root mean squared, distance is given by Eq (12).

12 $$ RMS = \sqrt{\frac{1}{n}\ {\displaystyle \sum_{i=1}^n{\left({C}_i-c\right)}^2}\ } $$

By using Eq (11), the scale factor *S* for the control points in *I*_*C*_ can be calculated as:

13 $$ S=\frac{\sqrt{2n}}{\sqrt{\ {\displaystyle {\sum}_{i=1}^n}{\left({C}_i-c\right)}^2\ }} $$

In the same way, the scale factor *S*′ for the control points in angiogram *I*_*A*_ can be calculated as:

14 $$ {S}^{\prime }=\frac{\sqrt{2n}}{\sqrt{\ {\displaystyle {\sum}_{i=1}^n}{\left({C}_i^{\prime }-{c}^{\prime}\right)}^2\ }} $$

a result, new two matrices containing normalized control points are computed using Eq (15) and (16).

15 $$ \tilde{C}={C}_T\times S $$

16 $$ {\tilde{C}}^{\prime }={C}_T^{\prime}\times {S}^{\prime } $$

And the new correspondent control points are shown as:

17 $$ \left\{{\tilde{C}}_i\ \leftrightarrow {\tilde{C}}_i^{\prime}\right\} $$

After normalization, the geometrical transformation function is computed using the normalized control points. We used the affine transformation matrix for this function, which has the following format:

18 $$ \tilde{T}=\left[\begin{array}{ccc}\hfill {t}_{11}\hfill & \hfill {t}_{12}\hfill & \hfill 0\hfill \\ {}\hfill {t}_{21}\hfill & \hfill {t}_{22}\hfill & \hfill 0\hfill \\ {}\hfill {t}_u\hfill & \hfill {t}_v\hfill & \hfill 1\hfill \end{array}\right] $$

When there are at least three normalized control points, the affine transformation function can be written as Eq (19).

19 $$ \left[\begin{array}{cc}\hfill {\tilde{C}}^{\prime}\hfill & \hfill 1\hfill \end{array}\right]=\left[\begin{array}{cc}\hfill \tilde{C}\hfill & \hfill 1\hfill \end{array}\right]\times \tilde{T} $$

where $\tilde{C}$ and ${\tilde{C}}^{\prime }$ are defined in Eq (15) and (16), respectively.

This equation can be simplified as:

20 $$ {\tilde{C}}^{\prime }=\left[\begin{array}{cc}\hfill \tilde{C}\hfill & \hfill 1\hfill \end{array}\right]\times {\tilde{T}}^{\prime } $$

where

21 $$ {\tilde{T}}^{\prime }=\left[\begin{array}{cc}\hfill {t}_{11}\hfill & \hfill {t}_{12}\hfill \\ {}\hfill {t}_{21}\hfill & \hfill {t}_{22}\hfill \\ {}\hfill {t}_u\hfill & \hfill {t}_v\hfill \end{array}\right] $$

Therefore, the affine transformation matrix of the normalized control points can be computed by Eq (22).

22 $$ {\tilde{T}}^{\prime } = {\left[\begin{array}{cc}\hfill \tilde{C}\hfill & \hfill 1\hfill \end{array}\right]}^{-1}\kern0.5em \times {\tilde{C}}^{\prime } $$

By calculating the above equation, all variables in ${\tilde{T}}^{\prime }$ are computed and $\tilde{T}$ are obtained.

Eventually, a proper correction should be done to bring the (normalized) transformation function $\tilde{T}$ to the original coordinate system. This step is called de-normalization and mathematically formulated in Eq (23).

23 $$ T = {N}^{-1}\times \tilde{T}\times {N}^{\prime } $$

with

24 $$ N=\left[\begin{array}{ccc}\hfill \frac{1}{S}\hfill & \hfill 0\hfill & \hfill 0\hfill \\ {}\hfill 0\hfill & \hfill \frac{1}{S}\hfill & \hfill 0\hfill \\ {}\hfill \overline{u}\hfill & \hfill \overline{v}\hfill & \hfill 1\hfill \end{array}\right]\kern0.75em \mathrm{and}\kern1em {N}^{\prime }=\left[\begin{array}{ccc}\hfill \frac{1}{S^{\prime }}\hfill & \hfill 0\hfill & \hfill 0\hfill \\ {}\hfill 0\hfill & \hfill \frac{1}{S^{\prime }}\hfill & \hfill 0\hfill \\ {}\hfill {\overline{u}}^{\prime}\hfill & \hfill {\overline{v}}^{\prime}\hfill & \hfill 1\hfill \end{array}\right] $$

By applying the transformation function *T*, the coronary artery from angiogram *I*_*A*_ is registered on the correspondent one in *I*_*C*_. The registered version of angiogram ${I}_A^{\hbox{'}}$ is formulated in Eq (25).

25 $$ {I}_A^{\prime }={I}_A\times T $$

As both arteries have been registered, they can be considered to be in the same plane, called image *I*_*R*_ in (*u*, *v*) plane, which contains both ${I}_A^{\prime }$ and *I*_*C*_.

After registration, the stenosis point from angiogram should be located on the correspondent artery in CTA.ost probably, the stenosis point from angiogram intersects with the coronary artery from CTA in *I*_*R*_. Otherwise, the stenosis point is estimated as follows. First, a proper thinning algorithm is used to extract the centrelines of both arteries in the registered plane, called ${I}_R^{\hbox{'}}$. Then, the points *p*_*k*_ and *p*_*l*_ are detected as the two nearest points to the stenosis point in ${I}_R^{\hbox{'}},$ such that the two arteries intersect at those points. Then, the length between the stenosis point and *p*_*k*_, and also the length between stenosis point and *p*_*l*_, for the artery from angiogram in ${I}_R^{\hbox{'}}$ are computed, called ${l}_1^{\hbox{'}}$ and ${l}_2^{\hbox{'}},$ respectively. In addition, the length of the centreline of the artery between two points *p*_*k*_ and *p*_*l*_ for the artery from CTA in ${I}_R^{\hbox{'}}$ is computed, called *L*. By calculating the ratio between the above lengths, the stenosis point is localized in the correspondent artery from CTA. It is formulated in Eq (26).

26 $$ {l}_1={l}_1^{\prime}\times \frac{L}{l_1^{\prime }+{l}_2^{\prime }}\kern3em {l}_2={l}_2^{\prime}\times \frac{L}{l_1^{\prime }+{l}_2^{\prime }} $$

**Figure 21:**
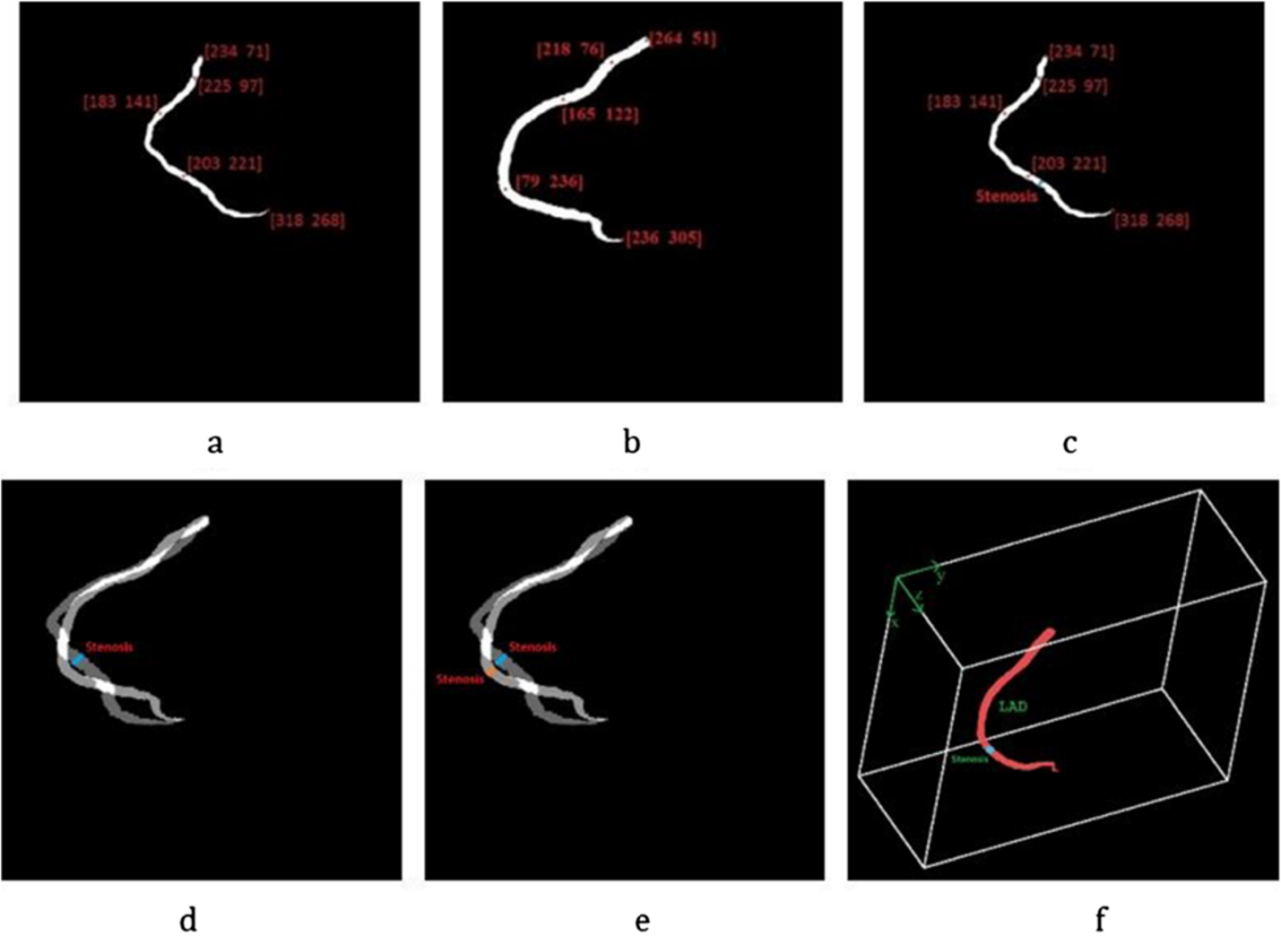
The result of coronary artery registration to locate 3D stenosis point on the CTA. **(a)** Segmented LAD from angiogram ***I***_***A***_ with candidate control points in 2D plane (*u*^'^, *v*^'^). **(b)** Segmented LAD from CTA with candidate control points, projected on 2D plane (*u*, *v*). **(c)** Marked stenosis position in the angiogram. **(d)** Registration result using the proposed method for both modalities shown on plane ***I***_***R***_. **(e)** Stenosis position estimated in the registration plane ***I***_***R***_ on coronary artery from CTA. **(f)** 3D position of stenosis in the original coordinate system (*x*, *y*, *z*).

After locating the stenosis point on *I*_*C*_, it is reconstructed in 3D. Since the 3D Cartesian coordinates (*x*, *y*, *z*) r each point in *I*_*C*_ are saved in the original 3D matrix of that artery, the stenosis point can be obtained by back projecting that point to the original 3D matrix. Some of the above steps are illustrated in Figure 21.

## Results and discussion

In this section, first the optimum values for parameters are reported and afterwards, the efficiency of each algorithm is evaluated. We obtained the dataset of angiogram and CT Angiography from the UiTM Medical Center. Five patients’ angiograms acquired with the PHILIPS Angiography device are used for the system configuration (training dataset) and 12 patients’ angiograms are used for the system analysis (test dataset), to evaluate the performance of the proposed method in this modality. The angiogram dataset includes 493angiograms of 12 different patients acquired in eight angles for each of patients. The format of them was 24-bit grayscale BMP in 512 × 512 pixels size. We randomly selected 60 angiograms (30 LCA and 30 RCA) from this as our dataset for this work, and manually created the ground truth for each. Also, four patients’ CTAs acquired with the SIEMENS 3D CT Angiography device are used in best diastole for the system configuration (training dataset) and 12 patients’ CTAs are used for the system analysis (testing dataset), to evaluate the performance of the proposed method in CTA modality. The images has the size of 512 × 512 pixels in the horizontal plane and about 450 slices of 0.75 mm for the *z* axis. The corresponding ground truth was created from the slices, as well. It is worth noting that all images in ground truth were validated accurately by the expert cardiac surgeon, cardiologist and radiologist. In this work, we applied the qualitative, and also quantitative methods such as accuracy, precision, sensitivity, specificity and the normalized sum of false detections values, to determine the performance of this work [34,35].

27 $$ Accuracy = \frac{T_p+{T}_n}{T_p+{T}_n+{F}_p+{F}_n} $$

28 $$ Precision = \frac{T_p}{T_p+{F}_p} $$

29 $$ Sensitivity = \frac{T_p}{T_p+{F}_n} $$

30 $$ Specificity = \frac{T_n}{T_n+{F}_p} $$

31 $$ {\upvarepsilon}_F = \frac{F_p+{F}_n}{2q} $$

where *T*_*p*_, *T*_*n*_, *F*_*p*_, *F*_*n*_ and *q* are defined:

*T*_*p*_, (True positive): the number of coronary artery pixels detected correctly.

*T*_*n*_, (True negative): the number of non-coronary artery pixels detected correctly.

*F*_*p*_ (False positive): the number of non-coronary artery pixels detected as coronary artery.

*F*_*n*_ (False negative): the number of coronary artery pixels not detected.

*q*: the quantity of all pixels inside an image.

### Setting the parameter values

Prior to accomplishing experiments regarding the overall performance assessment, suitable values in the proposed method’s parameters need to be determined. Therefore, we evaluated the effect of different values for each of the parameters and chose the best in each phase of this work.

#### Parameters in coronary artery segmentation and labeling in angiogram

**Figure 22:**
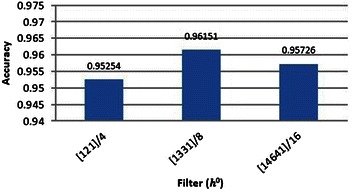
The effect of applying different filters *h* on the accuracy of the proposed method.

For coronary artery segmentation and labelling in angiogram, parameters filters (*h*^0^) and wavelet levels (*l*) parameters of the proposed algorithm were considered based on the accuracy of the proposed method for the main coronary artery segmentation. We examined these parameters on 20 different angiograms from the dataset, and finally calculated the average of each for optimization purposes. To obtain the best result in this method, we applied filters (*h*^0^) [1,2,1]/4, [1,3,3,1]/8 and [1,4,6,4,1]/16 on the dataset, and then calculated the accuracy for each. Results are shown in Figure 22.

As shown here, the best result was related to *h*^0^ = [1,3,3,1]/8.

**Figure 23:**
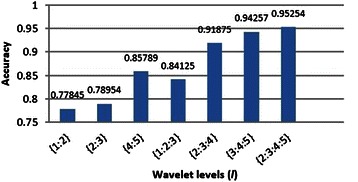
The effect of applying different wavelet levels on the accuracy of the proposed approach.

As explained before, thinner arteries are detected by smaller levels and thicker ones by higher levels. We applied the range of wavelet levels (*l* ⊆ {1,2,3,4,5}) for this research and examined their effects individually and as a whole. The obtained results are shown in Figure 23.

As demonstrated in Figure 23, the best result was obtained using wavelet levels *l* = {2,3,4,5}.

#### Parameters in coronary artery segmentation and labelling in CTA

**Figure 24:**
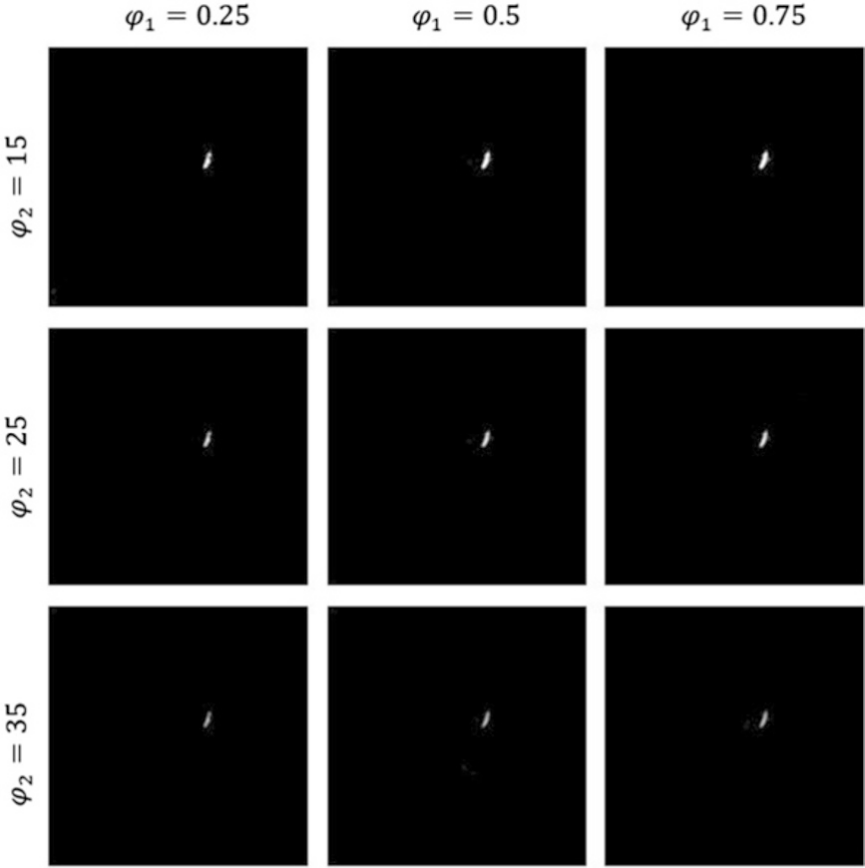
The effect of ***φ***_1_ and ***φ***_2_ parameters on coronary artery enhancement.

For mask construction, the slope and intercept parameters in Eq. (3) were obtained from the CTA DICOM info. In our dataset, they typically had “1” and “-1024” for slope and intercept, respectively. For coronary artery enhancement, two *φ*_1_ and *φ*_2_ parameters in Eq. (4) were obtained based on the dataset characteristics. We examined different value combinations on the vesselness measure function *v*. We considered the range of 0.25 to 0.75 for *φ*_1_ and the range of 15 to 35 for *φ*_2_ parameters. The effect of these combinations on the proposed Intersection Tracking method in a selected slice is shown in Figure 24, by considering a specific range of 1 ≤ *σ* ≤ 4 as the scale value. The value of *φ*_1_ controls the shape structure and should be less than 1, and *φ*_2_ establishes the particular effect associated with contrast robustness on artery enhancement. By choosing larger values of *φ*_2_, low-contrast items were usually disregarded in support of arteries along with considerable increase of contrast. Smaller values for this parameter, for instance, *φ*_2_ = 15, added more noise around the artery in the result in comparison with larger values. Nevertheless, a large value, for instance, *φ*_2_ = 35, caused considerable decline of the vesselness result, possibly for higher contrast arteries as shown in Figure 24.

The optimal values for *φ*_1_ and *φ*_2_ were determined by comparing the results of the proposed method on the slices of the dataset to the created ground truth. For this, the normalized sum of false detections (_*F*_) was calculated. Here, _*F*_ is an objective discrepancy measure that quantifies the change of the artery enhanced images acquired by utilizing various values associated with parameters *φ*_1_ and *φ*_2_, from the corresponding ground truth images. In this study, three values for *φ*_1_, *φ*_1_ = {0.25,0.5,0.75} and three values for *φ*_2_, *φ*_2_ = {15,25,35} were applied on the constructed dataset and the minimum discrepancy measure ε_*F*_ was calculated for all combinations. Based on the result of this experiment, the values 0.75 and 25 were selected for parameters *φ*_1_ and *φ*_2_, respectively.

### Experimental evaluation

Validation is usually a difficult, but essential phase for any coronary artery segmentation, labeling and registration techniques. With the exception of specific cases, such as centreline extraction, the main way to validate the results of coronary artery segmentation, labeling and registration in both CTA and angiography is by visual observation by an expert cardiologist and radiologist, or by comparison with the manually segmented case as the ground truth. We evaluated the proposed method in every phase of this work, as follows.

### Evaluation of main coronary artery segmentation and labeling in angiogram

In this section, we evaluated the capability of the main coronary artery segmentation of the proposed method on the dataset. First, we applied the proposed algorithm on 60 angiograms in the dataset, which included 30 LCA and 30 RCA. Then, we calculated the averages of accuracy, precision, sensitivity and specificity values for each.

As shown in Table 1, the average accuracy, precision, sensitivity and specificity values of the proposed method in LCA angiograms were 0.95153, 0.86812, 0.85897 and 0.97154, respectively. And as shown in Table 2, the average accuracy, precision sensitivity and specificity values of the proposed method in RCA angiograms were 0.96547, 0.92871, 0.89105 and 0.98154, respectively. With an emphasis on the capability of the proposed method, we compared our algorithm with a number of state-of-the-art coronary artery segmentation methods on the same dataset. To this end, the proposed methods by Khaleel *et al*. [4], Li *et al*. [9], Frangi *et al*. [30] and Bankhead *et al*. [26] were used for comparison. The result was summarized in Tables 1 and 2. As shown in these tables, all performance metrics such as, accuracy, precision, sensitivity and specificity for our method were much higher than the others for both LCA and RCA angiograms. Since Bankhead *et al*. [26] applied SWT method for vessel extraction in retina images, we also used their method here. They used the original SWT algorithm using the filter [1, 4, 6, 4, 1]/16, but as mentioned before, we modified this algorithm, especially for the filters and wavelet levels for main coronary artery segmentation in angiograms. Also, using the original SWT algorithm as used in Bankhead *et al*. [26], demonstrated that segmented arteries are thicker than the actual size and also increased *F*_*n*_ rate; because it could not detect the coronary artery pixels in some parts and as a result, it decreased the performance, especially in the sensitivity value as shown in Tables 1 and 2. This part of our proposed method was implemented in MATLAB R2014a. In the implementation, we used the original functions in the Image Processing Toolbox only, and because we aimed to decrease the running time, the additional compiled MEX code was not used. In Table 3, we compared the running time of the proposed method with some state-of-the-art methods. In this table, we calculated the running time for the methods proposed by Khaleel *et al*. [4], Frangi *et al*. [30], Li *et al*. [9] and also our proposed method using the same PC and conditions, while the running time for the method proposed by Zhou *et al*. [8] was obtained from their paper. As shown in Table 3, the proposed method needs lower computational time, less than 1 second, and it is a positive point for using this method in real-time systems. Therefore, we can consider it as a fast method for main coronary artery segmentation in angiograms. The results of manually labelling were evaluated by an expert cardiologist for 80 angiograms in our dataset. If the segmentation is done accurately, the proposed method for labelling will be robust to label all main coronary arteries in the angiograms.

**Table 1:** **The performance of the main artery segmentation of the left coronary artery (LCA) in the dataset**

Method	Accuracy	Precision	Sensitivity	Specificity
Khaleel *et al*. [4]	0.88277	0.34599	0.83859	0.88595
Li *et al*. [9]	0.90154	0.65875	0.83458	0.90423
Frangi *et al*. [30]	0.93795	0.52638	0.75311	0.95124
Bankhead *et al*. [26]	0.89772	0.62437	0.63692	0.91721
Proposed method	0.95153	0.86812	0.85897	0.97154

**Table 2:** **The performance of the main artery segmentation of the right coronary artery (RCA) in the dataset**

Method	Accuracy	Precision	Sensitivity	Specificity
Khaleel *et al*. [4]	0.89376	0.39544	0.86224	0.89624
Li *et al*. [9]	0.92483	0.64583	0.86843	0.91548
Frangi *et al*. [30]	0.96663	0.73889	0.82595	0.97703
Bankhead *et al*. [26]	0.91564	0.82587	0.64354	0.92547
Proposed method	0.96547	0.92871	0.89105	0.98154

**Table 3:** **Running time for coronary artery segmentation on dataset**

Method	Time (s)	PC	Programming language
Khaleel *et al*. [4]	1.48	Intel core i5, CPU 3.2 GHz, 8 GB RAM	MATLAB
Zhou *et al*. [8]	32	Pentium-IV, CPU 3.1 GHz, 4 GB RAM	MATLAB
Frangi *et al*. [30]	1.4	Intel core i5, CPU 3.2 GHz, 8 GB RAM	MATLAB
Li *et al*. [9]	12.3	Intel core i5, CPU 3.2 GHz, 8 GB RAM	MATLAB
Proposed method	0.68	Intel core i5, CPU 3.2 GHz, 8 GB RAM	MATLAB

#### Evaluation of the intersection tracking method for main coronary artery segmentation in CTA

The first part of the Intersection Tracking method evaluation is the seed point detection. As mentioned before, ostiums for every LCA and RCA are detected manually by seeking axial slices from top to bottom. Some algorithms have proposed automatic coronary artery seed point detection in CTA by considering the starting point from the aorta [36]. However, in some patients, there is a third coronary artery, Conus artery, which arises independently from the aorta. In this case, other algorithms failed to achieve the target seed points. Therefore, it is better to choose these points manually. As mentioned in the Intersection Tracking method, other seed points for LAD, LCX, are detected automatically. Therefore, the proposed method can be categorized as requiring minimal user-interaction by finding only the initial artery ostiums, as seed points.

Another part of the proposed method is bifurcation detection for removing sub-arteries. This process was evaluated using our datasets. According to the results, the LAD and LCX’s bifurcations were detected in all images in 12 datasets, but in one of them, only one of the diagonal’s bifurcation and also one of the septal’s bifurcation were missed. In addition, in two patients, the spurious bifurcations were detected. The spurious bifurcations were ignored because they did not have any intersecting region in consecutive slices. Therefore, we obtained legible results for bifurcation detection for 95.8% of our dataset.

**Figure 25:**
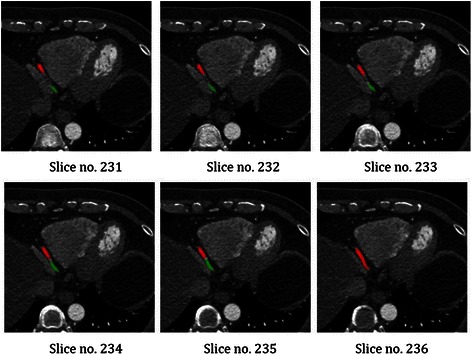
Some consecutive slices of the R-PLB artery for “Patient8” from the dataset. The algorithm failed to follow the end parts of the R-PLB artery (Missing parts are shown in green in each slice).

The last part of evaluating the Intersection Tracking method is the coronary artery segmentation. As mentioned in the proposed algorithm, every coronary artery can be segmented by tracking from the corresponding seed point and the following slices, until the artery ends. This method works based on the intersection between each slice and the next. This process was evaluated for each LM, LAD, LCX and RCA arteries on 12 datasets by comparing the results with the ground truth. Based on the comparison, the LM, LAD and LCX were segmented in most patients. But, part of the RCA failed at the end slices. The reason for this problem was the corresponding slices of the end of RCA, especially in parts of the R-PLB branch, were shown in previous slices but are not shown in the current slice. As shown in Figure 25, for example, parts of R-PLB for “Patient8” from the dataset was missed in slice no. 236. Even though they were visible in few previous slices, slices no. 231 to 235, but they did not have intersecting region with the current slice (slice no. 236). Detecting these parts in previous slices was enough to solve this problem. We added a backtracking step to the proposed algorithm to segment all parts of the artery, especially for those shown in previous slices.

The results of the comparison between the Intersection Tracking method on the dataset and the corresponding ground truth slices are shown in Table 4. In this table, the number of slices tracked using the proposed method was shown in comparison with the same slices in the ground truth.

**Table 4:** **A comparison between the results of artery segmentation using the intersection tracking method and the ground truth slices**

	NO. 1	NO. 2	NO .3	NO. 4	NO. 5	NO. 6	NO. 7	NO. 8	NO. 9	NO. 10	NO. 11	NO. 12
**LM**	13/14	12/12	13/13	14/15	12/12	13/13	15/16	9/9	13/13	14/15	13/13	16/16
**LAD**	112/114	105/108	112/113	113/114	108/110	117/120	129/132	97/100	105/108	124/127	119/122	131/133
**LCX**	213/215	198/200	178/185	210/216	195/202	200/208	224/230	186/189	185/191	216/218	207/210	231/237
**RCA**	138/146	114/119	124/125	136/140	130/138	139/142	147/152	119/125	110/117	127/130	132/137	146/149

**Figure 26:**
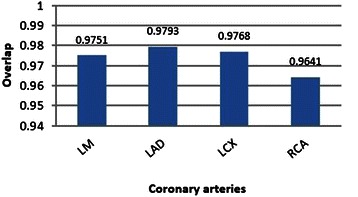
The result of overlap evaluation of the proposed intersection tracking method on LM, LAD, LCX and RCA arteries.

As shown in the table, the coronary arteries were tracked in most slices, and only failed in some, especially in those which had low contrast. The result of overlapping evaluation is shown in Figure 26.

**Figure 27:**
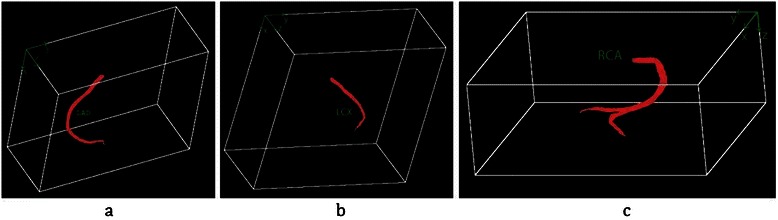
The result of coronary artery labeling and 3D reconstruction in CTA. Typical examples of LAD, LCX and RCA reconstructions are shown in **(a)**, **(b)**, and **(c)**, respectively.

The next step of this phase is 3D coronary artery reconstruction and labelling. As discussed before, coronary arteries are segmented as cross sectional in each slice and kept in different 3D matrices using the proposed Intersection Tracking method. It means that for all LM, LAD, LCX and RCA, 3D matrices *M*_*LM*_, *M*_*LAD*_, *M*_*LCX*_ and *M*_*RCA*_ are constructed respectively, which preserve each pixel of the corresponding artery in the 3D coordinate *R*^3^ domain. For 3D reconstruction and visualization, the OPENGL library in VC++ program was applied. A typical example of the labeled arteries for “Patient12” from the dataset is shown in Figure 27, where the various branches were labelled separately.

**Figure 28:**
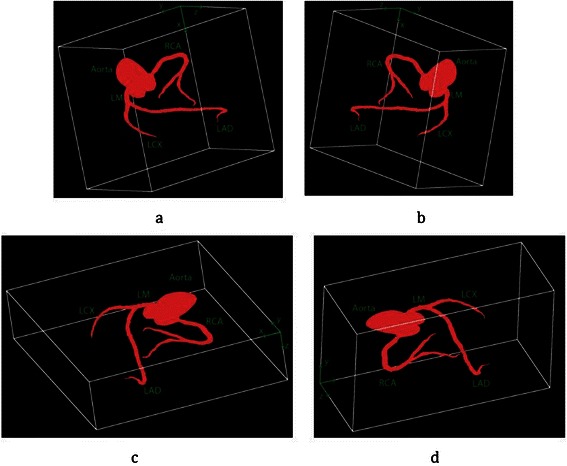
3D reconstruction of whole coronary arterial tree in CTA using the Intersection Tracking method. **(a)** - **(d)** Results of four different views.

As all arteries are preserved in different matrices, it is possible to show all of them together. This type of visualization has many advantages for cardiologist and cardiac surgeon. All matrices *M*_*LM*_, *M*_*RCA*_, *M*_*LAD*_, *M*_*LCX*_ and *M*_*Aorta*_ can be merged together, to create one 3D matrix for the whole arterial tree. The same method was used for 3D reconstruction from the merged matrix. The whole coronary arterial tree for “Patient12” is visualized in Figure 28, in four different views. As discussed before, *x* and *y* are the coordinates of every segmented slice and *z* is the slice number in CTA, from top to bottom.

Our method was compared with the one and only studied on coronary artery labeling in CTA [14], where arteries were labeled in separate phases after the segmentation, centreline extraction and registration steps. In [14], first the arteries were segmented and arteries’ centreline was extracted. Then, the point-set registration method in [15] was applied for identifying main branches. However, in our proposed Intersection Tracking method, the segmentation and labelling were done together, at the same time and there was no need for other processes, such as centreline extraction or prior image registration. Therefore, it is faster and also more accurate in comparison with [14]. The overall overlap of their labelling was 91.41%. In our study, the overlap amount is related to the overlap in segmentation, which means that, if coronary artery segmentation is done accurately, the labelling will be done completely without any error as well. Since the overall segmentation amount of the proposed work is 97.38%, it is obvious that the overall overlap for labelling has the same value.

#### Validation of CTA and angiography registration and evaluation of the 3D position of stenosis

**Figure 29:**
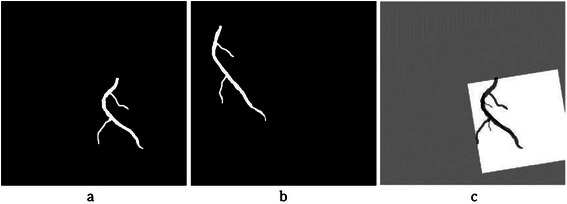
The results of registration without control point for two selected images. **(a)** Image *A* from CTA as the fixed image. **(b)** Image B from angiogram as the moving image. **(c)** The result of applying affine transformation without control point.

In this experiment, the result of the proposed method for coronary artery registration from two modalities is validated. Then, the 3D position of the stenosis of the coronary artery is evaluated. For the registration, it is not possible to evaluate the accuracy of real data, such as the heart, using a mathematical criterion. However, the specialist can visually validate them. For this, first the results of applying conventional methods, which did not use control points, on two correspondent images *A* and *B* from the dataset, are illustrated in Figure 29.

**Figure 30:**
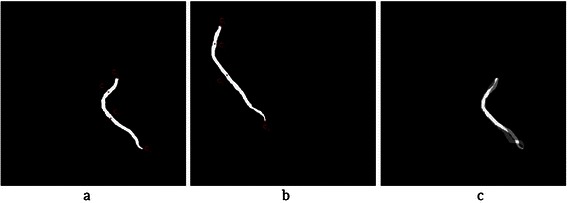
The result of the proposed method for main coronary artery registration with control points. **(a)** Main coronary artery segmented and labeled from CTA including control points. **(b)** Main coronary artery segmented and labeled from the angiogram including control points. **(c)** The result of applying the proposed method.

As shown in this figure, the coronary arteries were not registered well without control points. The result of extracting {*C*_1_,*C*_2_,*C*_3_} and $\left\{{C}_1^{\prime },{C}_2^{\prime },{C}_3^{\prime}\right\}$ as the two sets of the correspondent control points in *A* and *B*, respectively, and also applying the proposed method for registration, are illustrated in Figure 30.

The specialist found that the result of the proposed method for registration was much better than the conventional method. As shown in this figure, the control points in the affine transformation are registered exactly and most parts of the correspondent arteries are registered. It is worth noting that if the control points in both modalities could be registered exactly and the correspondent arteries could be registered exactly, the 3D position of the stenosis lesion would be more accurate. In addition, if the distance between the stenosis point and its two nearest control points is small, the result would be more accurate.

To evaluate the 3D position of the stenosis point on the coronary artery, 10 patients were chosen from the database as the dataset for this part. The criterion for choosing these patients were based on the fact that their stenosis points could be manually detected by the specialist in both CTA and angiography modalities. In this manner, coronary arteries and their stenosis positions on the CTA modality were known before. Therefore, the mean 3D distance between the stenosis point on the result of the proposed method and the stenosis point detected by the specialist in CTA, was calculated for each patient. In fact, the mean distances should be ideally null. It is worth noting that from the dataset, three patients had stenosis lesion in the right coronary artery (RCA) and seven patients had stenosis lesion in the left coronary artery (LCA); including 3 patients in LAD, 2 patients in LCX and 2 patients in LM arteries. Table 5 shows the results of the evaluation using the above dataset.

**Table 5:** **The result of the 3D stenosis position evaluation**

Patient	Stenosis position	Distance
1	*RCA*	≈0.71 cm
2	LCA, LCX	≈1.04 cm
3	RCA	≈0.85 cm
4	LCA, LAD	≈1.05 cm
5	LCA, LAD	≈0.68 cm
6	RCA	≈0.62 cm
7	LCA, LAD	≈0.94 cm
8	LCA, LM	≈0.18 cm
9	LCA, LCX	≈0.89 cm
10	LCA, LM	≈0.23 cm

The mean value and standard deviation were 7.19 *mm* and 3.07 *mm*, respectively. The main reasons for these distances were related to the nature of the heart, whereby the heart is a non-solid organ and also is beating. Therefore, there are dissimilarities between the coronary arteries of the two different modalities.

Another problem in the conventional registration algorithms is that they are time consuming; the registration part only takes about 1 min. However, in the proposed method, the registration part using the control points took about 0.31 s. This specific computational time was based on a common personal computer set up with Intel core i5 (CPU 3.2 GHz) and 8 GB RAM, and the program being created in MATLAB R2014a. Furthermore, this computational time can be decreased further by utilizing better state-of-the-art computer. It is worth noting that the proposed method provides further information, which can complement the angiogram and CTA. By using this method, it is possible to visualize both the correspondent coronary arteries from angiogram and CTA together. This kind of information assists the cardiac surgeon and cardiologist to make a decision regarding whether an artery needs to be dilated or not.

## Conclusions

A new method is proposed for coronary artery registration in both CTA and angiography modalities to provide the 3D position of the stenosis lesion diagnosed in an angiogram. The result has benefits for CAD diagnosis and treatment. Tests using the dataset demonstrated that the proposed method aided the specialists to find the location of the stenosis lesion and also determine the visual relationship between the correspondent coronary arteries. To the best of our knowledge, there is no hybrid device for both CTA and angiography modalities, yet. In addition, based on the literature, no algorithm has been proposed for registering these two modalities in order to obtain the 3D position of the stenosis point. Therefore, the proposed method can be seen as a new contribution. The aim of the proposed algorithm is applicable and portable for common personal computers, also with respect to the standard medical acquisition methods. In the future, we intend to perform a comprehensive research on stenosis detection in angiography images and apply the proposed method to automatically mark the stenosis point in the angiogram.

## Abbreviations

CTA: Computed tomography angiography
CAD: Coronary artery disease
RCA: Right coronary artery
LCA: Left coronary artery
LCX: Left circumflex coronary artery
LAD: Left anterior descending coronary artery
DWT: Discrete wavelet transform
SWT: Starlet wavelet transform
HU: Hounsfield unit
RMS: Root mean squared
